# The Use of Vitamins and Coenzyme Q10 for the Treatment of Vascular Occlusion Diseases Affecting the Retina

**DOI:** 10.3390/nu12030723

**Published:** 2020-03-09

**Authors:** Beatriz Fernández-Vega, Javier Nicieza, Ana Álvarez-Barrios, Lydia Álvarez, Montserrat García, Carlos Fernández-Vega, José A. Vega, Héctor González-Iglesias

**Affiliations:** 1 Instituto Oftalmológico Fernández-Vega, Avda. Dres. Fernández-Vega, 34, 33012 Oviedo, Spain; mgarciadiaz@fio.as; 2 Instituto Universitario Fernández-Vega (Fundación de Investigación Oftalmológica, Universidad de Oviedo), 33012 Oviedo, Spain; uo245562@uniovi.es (A.Á.-B.); l.alvarez@fio.as (L.Á.); c.fernandezvega90@gmail.com (C.F.-V.); 3 Departamento de Morfología y Biología Celular, Grupo SINPOS, Facultad de Medicina y Ciencias de la Salud, Universidad de Oviedo, Avda. Julián Clavería, 6, 33006 Oviedo, Spain; javega@uniovi.es; 4 Hospital Universitario de Cabueñes, Los Prados, 395, 33394 Gijón, Spain; jniciezab@telecable.es; 5 Facultad de Ciencias de la Salud, Universidad Autónoma de Chile, Calle Magdalena Vicuña 1281, 8900000 Santiago de Chile, Chile

**Keywords:** Vascular diseases, retina, visual field defects, dietary supplementation, vitamins, coenzyme Q10

## Abstract

Nutritional supplementation with antioxidants and vitamins is widely recommended in the treatment of vascular disorders affecting the retina, although there is insufficient evidence on its effectiveness. The vitamin-like compound coenzyme Q10 (CoQ10) is a nutritional supplement of current interest to treat neurodegenerative diseases. Here, we report a retrospective clinical case series study of 48 patients diagnosed with retinal vascular diseases, including non-arteritic ischemic optic neuropathy (NAION), retinal artery occlusion (RAO), and homonymous hemianopia or quadrantanopia following stroke, treated with oral supplementation with CoQ10 (100 mg per day) and vitamins. Patient follow-up was performed using the Humphrey field analyzer and 30-2 testing algorithm to determine the visual field index (VFI) and progression rates. All treated patients showed positive VFI progression rates per year: +11.5 ± 15% for NAION patients (*n* = 18), +22 ± 17% for RAO patients (*n* = 7), +9.3 ± 10.5% for hemianopia/quadrantanopia patients (*n* = 10), and +11 ± 21% for patients with other conditions (*n* = 13). The interruption of CoQ10 supplementation in one patient resulted in a pronounced decrease of the VFI, which was partially recovered when treatment was restored. This study supports the role of CoQ10 as a nutritional therapeutic agent for vascular diseases affecting the retina. Owing to decreased VFI after interruption of CoQ10, its beneficial effects may be reversible.

## 1. Introduction

Retinal dysfunctions caused by vascular disorders resulting in sudden visual loss are currently very prevalent. Vascular diseases can affect the blood flow to the optic nerve, the ophthalmic artery, and the retinal vein, or even produce retrochiasmal lesions of the visual pathway following cerebral insult. Among them, ischemic optic neuropathy (ION) is a spectrum of ocular diseases affecting the optic nerve owing to the interruption of blood flow supplied by the posterior ciliary arteries (anterior ION) or the pial capillary plexus (posterior ION) [1]. The most common anterior ION is the non-arteritic (NAION) form, presenting acute, unilateral, and painless loss of central and/or peripheral vision that can progress over several hours or days, mainly affecting patients older than 50 years [2]. On the other hand, retinal artery occlusion (RAO) is an embolic or thrombotic occlusion of either the central or branch ophthalmic artery (CRAO, BRAO), resulting in ischemia of the retina and visual loss. The retinal artery occlusion, mainly unilateral, may be transient and last for only a few seconds or minutes if the blockage breaks up and the flow restores, occurring mostly in male patients over 60 years [3,4,5]. Moreover, damage to the occipital, parietal, or temporal lobes caused by cerebral insults, including stroke, trauma, tumor, or brain surgery, results in severe visual field defects. Lesions affecting postchiasmal afferent nerve pathways generally produce homonymous visual field loss, which can be a hemianopia (one-half of an eye’s visual field) or quadrantanopia (one-fourth of an eye’s visual field) depending on the location of the lesion, with high impact upon the patient’s quality of life [6,7].

Vascular diseases affecting the eye are multifactorial in origin, with many risk factors contributing to their onset. Systemic risk factors include nocturnal arterial hypotension, arterial hypertension, diabetes mellitus, hyperlipidemia, ischemic heart disease, blood loss, atherosclerosis, sleep apnea syndrome, cardiovascular disorders, tobacco smoking, obesity, atrial fibrillation, hyperhomocysteinemia, varicose veins, and coagulation disorders (activated protein C resistance or factor V Leiden mutation), among others [3,8,9,10,11]. Ocular risk factors include elevated intraocular pressure, glaucoma, a small and crowded disc, or optic disc drusen that can influence the optic nerve head blood supply [9,12].

Current therapies are unable to predictably alter the natural history of these diseases, with limited efficacy to improve vision. During the acute event, ocular massage contributes to improve retinal perfusion, while lowering intraocular pressure, paracentesis, or hemodilution can increase the vascular supply. The use of vasodilators, thrombolytics, or a high concentration of oxygen is also recommended. The approach is mainly based on clinical evaluation and reduction of as many risk factors as possible to diminish the risk of any further episodes. However, no definitive standard treatment options exist due to insufficient high-quality evidence-based research [1,3,13].

Prescription of nutritional supplements, including vitamins, antioxidants, and omega-3 fatty acids, is widely recommended in the treatment of visual field defects of vascular origin [14,15]. However, there is a lack of standard pharmacological treatments or broadly accepted nutritional recommendations owing to the absence of evidence of effectiveness. One nutritional supplement of potential interest for the treatment of retinal dystrophies is coenzyme Q10 (CoQ10) [16,17,18,19,20]. CoQ10, a vitamin-like compound, is a physiological component of the electron transport chain, which maintains the mitochondrial membrane potential, supports adenosine triphosphate (ATP) synthesis, and functions as an antioxidant protecting neurosensorial cells [21].

In a seminal case report [22], we documented the clinical findings and management of a case of occipital lobe infarction with homonymous quadrantanopia in a patient treated with vitamins and coenzyme Q10, with significant visual field recovery 10 years after the cerebral insult. This documented improvement in the visual field of the treated patient, whose visual recovery was considered unlikely after more than 6 months post-injury, may be a sign of therapeutic efficacy. To address this observation, in the current work we present a retrospective case series study of vascular occlusion affecting the retina, including NAION, RAO, homonymous hemianopia or quadrantanopia, and optic nerve dystrophies, inter alia, treated with vitamins and CoQ10, observing remarkable improvements in the visual field, which supports the use of this coenzyme as a nutritional therapeutic agent for retinal diseases of vascular origin.

## 2. Materials and Methods 

Cases of patients suffering vascular diseases affecting the retina and treated with vitamins and CoQ10 supplements were retrospectively reviewed. The study adheres to the tenets of the Declaration of Helsinki on Biomedical Research Involving Human Subjects, and was approved by the Clinical Research Ethics Committee (Reference 254/19) at Principality of Asturias (Oviedo, Spain). All subjects gave their informed consent for inclusion of their clinical case in the study. Specifically, 18 patients diagnosed with NAION, 10 patients with homonymous hemianopia (or quadrantanopia) caused by cerebral stroke, 7 patients with RAO, and 13 patients with other conditions, including optic nerve atrophy, cone dystrophy, retinitis pigmentosa, and retinal vascular occlusion, were recruited at the Ophthalmological Institute Fernández-Vega. A group of patients with other diseases was established to simplify the study, which included optic nerve atrophy, retinitis pigmentosa, retinal vascular occlusion, etc. Patients were diagnosed by indirect ophthalmoscopy, visual field analysis, determination of nerve fiber layer thickness and macula by optical coherence tomography (OCT), visual acuity, evoked potentials, examination of the papilla and anterior segment, fluorescein angiography (FA), best-corrected visual field acuity, and neurological study, including magnetic resonance imaging, when needed. No subjects involved in this study presented with other relevant ocular pathologies. Once each patient was diagnosed, systemic treatment with vitamins and antioxidants was prescribed, mainly consisting of the following: Active complex® Q10 Gold 100 mg (Pharma Nord, Vejle, Denmark), 1 tablet at lunch in all months, following manufacturer’s dosage guidelines; Hidroxil® (vitamins B12, B6, and B1; Almirall, Barcelona, Spain), 1 tablet at breakfast, lunch, and dinner in alternate months; Acfol (Italfarmaco, Cinisello Balsamo, Italy), 1 tablet at breakfast in alternate months; Cebrolux 800 (Bausch & Lomb, New York, NY, USA), 1 dose at lunch for 3 months and 1 resting month; Visan (Théa laboratoires, Clermont-Ferrand, France), 1 tablet at breakfast in the Hidroxil and Acfol resting month; and, for some patients, Adiro 100 or 300 (Bayer, Leverkusen, Germany), 1 tablet before meals. Specific treatments and dosages, depending on the main active ingredient, for each patient are included in Supplementary Table S1. Active complex® Q10 Gold 100 mg contains 100 mg of CoQ10 and 25 mg of vitamin C per tablet, while the active ingredient of Adiro 300 is acetylsalicylic acid (100 or 300 mg per tablet) and it is used for stroke (antiplatelet). The active ingredients of Hidroxil include vitamins B1 (250 mg thiamin), B6 (250 mg pyridoxine), and B12 (500 μg cyanocobalamin) (all amounts per tablet). The active ingredient in Acfol is folic acid (5 mg per tablet). Visan nutritional supplement contains trace elements (5 mg of zinc, 0.5 mg of copper, 27.5 μg of selenium, and 1 mg of manganese), vitamins (200 μg of A, 40 mg of C, 6 mg of E, 0.55 mg of B1, 0.7 mg of B2, 8 mg of B3, 0.7 mg of B6, and 1.25 μg of B12), 1.5 mg of lutein, 0.15 mg of zeaxanthin, 2.5 mg of glutathione, 25 mg of flavonoids, and low levels of coenzyme Q10 (2.5 mg) in each tablet. Cebrolux 800 dietary supplement contains 163 mg of choline and additionally 20 mg of vitamin C, 3.3 mg of vitamin E, 0.264 mg of vitamin A, and 0.4 mg of vitamin B6.

None of the patients received restorative training, optical aids, or compensatory training. Patient follow-up was carried out using the Humphrey field analyzer (HFA) instrument (Zeiss Iberia, Madrid, Spain) and 30-2 Swedish interactive threshold algorithm (SITA) standard. This algorithm uses a mathematical model to estimate threshold values for each point based on responses to stimuli. The visual field index (VFI), reflecting retinal ganglion cell loss and function, and the mean deviation (MD) in decibels (dB), representing retina sensibility, were obtained to evaluate the percentage of the remaining visual field of the right and left eyes. The program of the instrument provides the progression rate along the follow-up of each patient obtained by a linear regression analysis of VFI (or MD) with the corresponding slope and level of significance. Patient follow-up and examination were highly variable, depending on the severity of the disease. Upon initial examination and following treatment prescription, the patients were initially revised from 2–6 months to yearly follow-up. Comparisons between the initial and final values of VFI (%) and MD (db) of each group of patients were carried out using the Wilcoxon matched-pairs test (GraphPad InStat version 3.0; San Diego, CA, USA), with a p-value less than 0.05 considered statistically significant. Demographic and clinical characteristics of patients are reported in Table 1 and Supplementary Table S1, in which the disease, sex, affected eye, age at diagnosis and at initiation of CoQ10, and vitamin supplementation treatments are indicated, along with relevant ocular and systemic diseases. Clinical history of patients included in this study covers a range from 5 to 203 months, with average follow-up of 43 ± 41 months. 

## 3. Results

The studied cohort, from a Spanish population, consisted of 48 patients diagnosed with NAION (18), homonymous visual field loss (hemianopia or quadrantanopia) following stroke (10), RAO (7), or other conditions (13) (Table 1). The population-averaged age at diagnosis was 57 ± 16, with an age range of 15–85 years. Among the patients, 46% were female, but with high variability depending on the disease. The age ranges of NAION and RAO were 50–81 and 48–83 years, respectively, indicating that both are late-onset disorders.

Follow-up of each condition was depicted by VFI and MD parameters of each eye, obtaining the VFI progression rate per year in the right and left eyes, at a 95% confidence level. Table 2 shows the NAION cases, including the number of months of follow-up, the initial (first examination) and final (last examination) VFI and MD in % and dB, respectively, and the VFI progression rate of both eyes in % per year, even if only one eye was affected in each case. Superscripts in the table show the date of CoQ10 treatment initiation, i.e., at month 0 upon initial examination or in the corresponding month. VFI progression rates were highly variable, with no noticeable changes in non-affected eyes. However, the affected eyes showed progression rates of +0.2 ± 0.6% per year (NAION18 left eye, no relevant differences), +2.1 ± 1.9% per year (NAION01 left eye), and +23.2 ± 14.5% (NAION13 left eye). Three patients, upon initial examination, presented VFI of 0% (NAION02), 4% (NAION14), and 3% (NAION17), improving to 21%, 20%, and 73%, respectively, to date. The average of progression rate reached +11.5 ± 15% per year considering all patients. Most of the patients experienced remarkable enhancement of the visual field at a 95% confidence level after the prescription of vitamins and CoQ10 treatment. 

Characteristics of patients diagnosed with homonymous hemianopia or quadrantanopia following stroke are summarized in Table 3. Both eyes were affected in all patients and the VFI progression rate was highly heterogeneous depending on the case, with an average progression rate reaching +9.3 ± 10.5% per year, considering all patients in this subgroup. Follow-up of individual patients ranged from 6 (Stroke02) to 203 (Stroke03) months, with progression rates of +0.6 ± 0.9% (right eye of Stroke01) to +33.3 ± 10.7% (right eye of Stroke10). Once again, most of the patients experienced notable enhancement of the visual field at a 95% confidence level after the prescription of vitamins and CoQ10 treatment. The Stroke07 case must be highlighted, considering that upon initial examination the patient did not present specific signs of visual pathway lesions, while in the third month a cerebral ictus with peripheral alteration was observed with VFI of 88% (right) and 80% (left) and almost total recovery after CoQ10 and vitamin treatment, with progression rates of +3.3 ± 3.8% and +4.9 ± 4.6% in right and left eyes, respectively (95% confidence level). 

**Figure 1:**
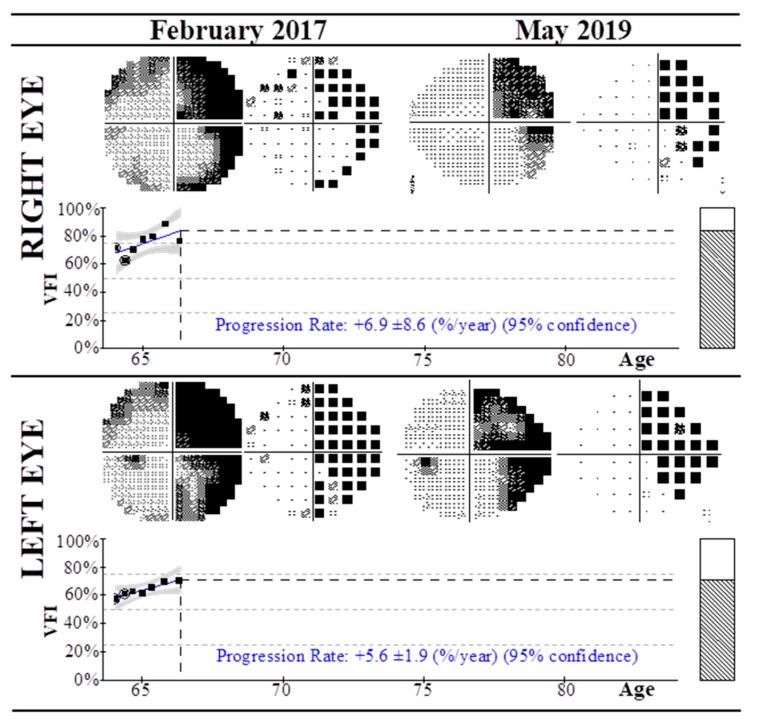
Visual field test using the Humphrey field analyzer and 30-2 algorithm of a case of right homonymous incomplete hemianopia after left occipital lobe stroke (Stroke06) from February 2017 to May 2019. In 2017 the patient was treated with CoQ10 and vitamins until May 2019, with progressive improvement of the visual field index in both eyes. Progression rates at 95% confidence level are shown.

Figure 1 exemplifies one of the selected cases (Stroke06) of a patient diagnosed with right homonymous incomplete hemianopia after left occipital lobe stroke, showing the visual field test using the HFA and 30-2 algorithm from February 2017 to May 2019. In 2017 the patient presented with right homonymous hemianopia with VFI of 72% and 58% in the right and left eyes, respectively (see Table 3). At that time, the patient was prescribed Active complex® Q10 Gold 100 mg, containing 100 mg of CoQ10 and 25 mg of vitamin C, in alternate months (1 tablet at lunch, following manufacturer’s dosage guidelines), in addition to Hidroxil, Acfol, and Visan supplements. At two-year follow up (2019), the VFI of the right eye had a slight improvement of the visual field (76%), and a remarkable improvement was observed in the VFI of left eye (from 58% to 71%), with progression rates of +6.9 ± 8.6 and +5.6 ± 1.9 per year, respectively.

**Figure 2:**
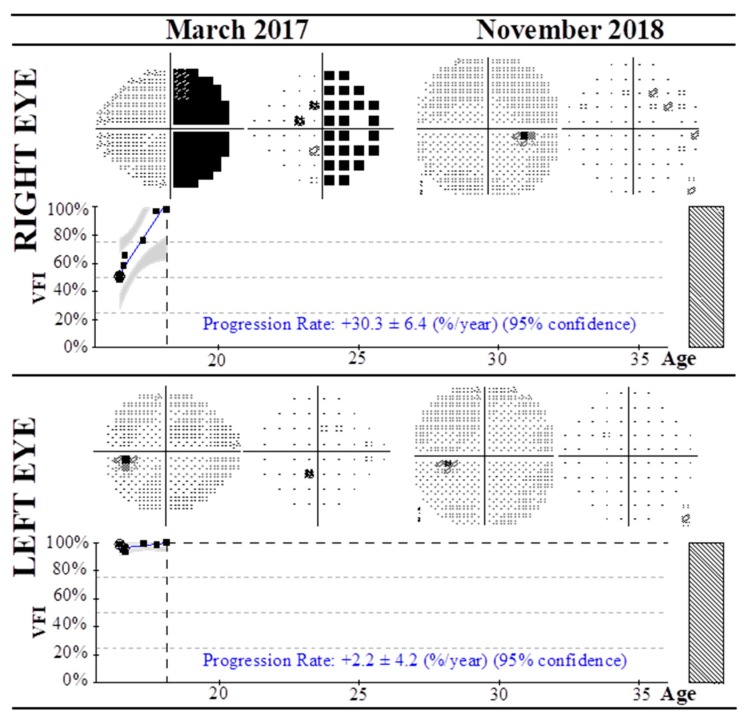
Visual field test using the Humphrey field analyzer and 30-2 algorithm of a case of right hemianopia of unknown origin (Stroke 04) from March 2017 to November 2018. After four months of the acute event the patient was treated with CoQ10 and vitamins to date, with progressive improvement of the visual field index in the affected right eye. Progression rates at 95% confidence level are shown.

Additionally, Figure 2 depicts a very interesting case of a young patient diagnosed with right hemianopia of unknown origin (Stroke04) affecting the right eye, with initial VFI of 51%. The patient also presented RPE alteration and myopia with growth hormone prescription from 2012 to 2015. During the first 4 months of follow-up, the VFI remained almost constant, from 51% to 58%. In the fourth month, oral treatment with vitamins and CoQ10 was prescribed, and a pronounced progression of VFI was observed, with a rate of +30.3 ± 6.4% for the affected right eye and 99% of the visual field in the 20th month of follow-up.

RAO patients treated with CoQ10 and vitamins from the first month following the acute event presented the most dramatic vision improvement, as shown in Table 4. The average progression rate was +22 ± 17% per year considering all patients. Follow-up of individual patients ranged from four (RAO03) to 53 (RAO07) months, with progression rates of +3.0 ± 5.3 (left eye of RAO07) to +49.4 ± 42.9 (right eye of RAO02). Two patients, upon initial examination, presented VFI of 0% (RAO01 and RAO02), improving to 75% and 46%, respectively.

**Figure 3:**
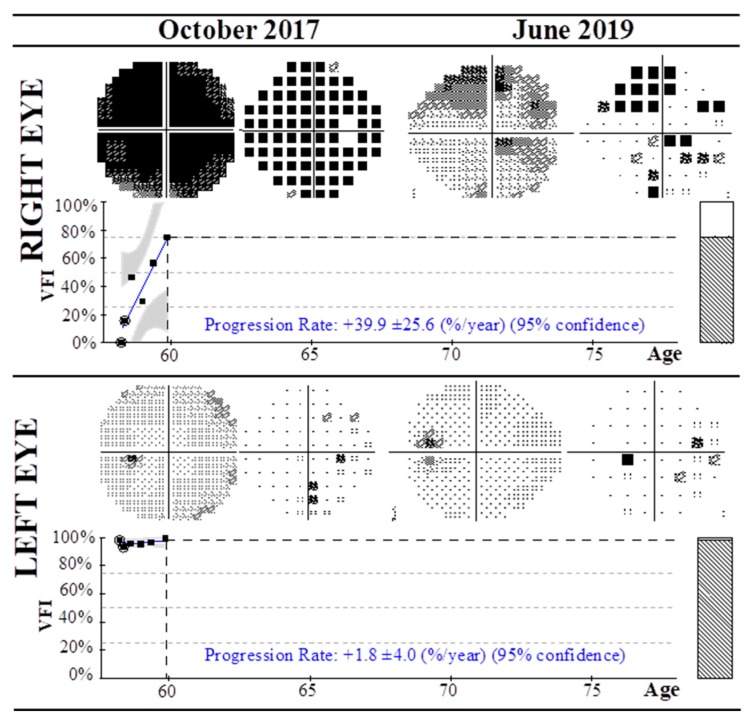
Summary of visual field test with Humphrey field analyzer and 30-2 algorithm of a patient diagnosed with CRAO affecting the right eye (RAO01) from October 2017 to June 2019. From 2017 to date the patient was treated with CoQ10 and vitamins, with progressive improvement of the visual field index in both eyes. Progression rates at 95% confidence level are shown.

Specifically, VFI evolution of the RAO01 case (patient diagnosed with CRAO) from October 2017 to June 2019 is summarized in Figure 3. In 2017 the patient presented a VFI of 98% in the non-affected left eye, while this index reached 0% in the affected right eye without any sign of vision. The patient was then treated with CoQ10 and vitamin supplementation, and after nine months of follow-up the patient experienced pronounced improvement of VFI to 75% in the right eye (see Table 4). The VFI progression rates per year reached +39.9 ± 25.6 and +1.8 ± 4.0 in the right and left eyes, respectively. 

The cases of patients with different conditions affecting the vascularity of the retina (*n* = 13) are collected in Table 5. These include optic neuritis, optic nerve atrophy, neuroretinitis, cone dystrophy, retinitis pigmentosa, retinal vascular occlusion, and two of unknown etiology. The follow-up covers a period of 14–110 months, with an average progression rate reaching +11 ± 21% per year considering all patients. Most of the patients presented with both eyes affected, with minimum VFI detected of 0% and 2% (right and left eye of OC04), 5% and 11% (right and left eye of OC10), and 12% and 19% (right and left eye of OC09). Specifically, patient OC04 experienced almost complete recovery in the VFI of the left eye, with an increase from 2% to 91% (95% confidence level). The OC09 case should be highlighted: initially not treated with CoQ10, and with constant VFI from the first month (12% left and 19% right eye) to the 47th (9% left and 25% right eye), after the 47th month, the patient was prescribed CoQ10 and vitamin supplementation, and experienced a remarkable recovery of VFI to 49% in both eyes, with overall average progression rates of +0.0 ± 3.7% and +0.4 ± 2.7% for the right and left eyes.

Finally, Table 6 shows the *p*-values obtained in the comparisons between initial and final VFI and MD of each group of patients, using the Wilcoxon matched-pairs test. As can be observed, all comparisons within each group show statistically significant differences, indicating an overall improvement of the visual field. 

## 4. Discussion

This case series study shows clinical outcomes of treatment with CoQ10 and vitamins in patients with retinal diseases of vascular origin in different periods from 2009 to 2019. Visual field defects are common after vascular occlusions, stroke, trauma, tumor, brain surgery, and demyelinating lesions, with a high impact on daily activities, with poor mobility, collisions, impaired reading and driving skills, and increased dependence and disability [23,24]. The prognosis of retinal diseases of vascular origin is uncertain and adverse with current knowledge. For example, RAO symptoms will remain stable or worse over time unless the patient has a cilioretinal artery, which lessens the chances of damage [25], while the clinical course of NAION generally remains stable, with most cases showing no noticeable improvement or deterioration over time [26]. Moreover, stroke following homonymous hemianopia (or quadrantanopia) has a poor prognosis, with unlikely spontaneous recovery [27]. 

Usually, visual field defects of vascular origin undergo recovery in the first days after the insult, probably mediated by removal of the edema and concomitant restitution of surrounding non-infarcted penumbral tissue, with uncertain further amelioration of the disease. In NAION or RAO, spontaneous improvement of visual acuity is not unusual during the first weeks following the event, although significant improvement in the visual field seems to occur less commonly than improvement in acuity [28]. Moreover, approximately 60% of patients diagnosed with hemianopia can experience spontaneous improvement in the visual field, usually in the first 10 days after brain injury and decreasing progressively with every successive month, with less than 10% of patients recovering their full field [29]. Therefore, the recovery is variable, depending on the degree of neuronal death and the removal of the initial effects of the acute injury [13,30]. In our case series, the improvement in patients’ visual field began to occur in most cases more than two months after the lesion, with important recovery during follow-up, and therefore was not likely a result of spontaneous recovery. It must be highlighted that the initiation date of treatment varied among patients, as can be observed in Table 2, Table 3 and Table 4, since many patients were referred from other hospitals and it is not possible to determine the exact period of vision loss. These differences in the initiation of CoQ10 supplementation may have an unknown impact on VFI outcomes.

Visual field improvement implies notable changes in mean and pattern deviations in Humphrey VFI, with varying rates of recovery depending upon the individual. In this case series we obtained the natural history of visual field recovery of 48 patients at different stages of disease and with various treatment periods. Vascular diseases affecting the retina are initially addressed to identify the vascular etiology, modify risk factors to prevent reoccurrence, improve retinal perfusion, lower intraocular pressure, increase the vascular supply using vasodilators, and initiate early and intensive rehabilitation therapy to allow better clinical outcomes and disability improvement [13,31]. There is no consensus on gold-standard treatment due to inadequate evidenced-based research. Currently, vitamin and antioxidant supplements are widely used without broadly accepted nutritional recommendations, owing to the lack of evidence of effectiveness. Recently, CoQ10 supplementation has been proposed for the treatment of retinal dystrophies [20]. In line with this, in most of the cases included in this study we observed amelioration in the visual field following CoQ10 and vitamin supplementation along each respective follow-up, with average progression rates of +13 ± 16% per year (Table 2, Table 3, Table 4 and Table 5). Interestingly, an additional comparison of the initial and final VFI and MD values within each of group of patients (NAION, Stroke, RAO, and OC) showed statistically significant differences, confirming the beneficial effects of CoQ10 and vitamin treatment in the improvement of visual field progression (Table 6).

Among the studied cases, the patients diagnosed with RAO (*n* = 7) experienced the highest increase of VFI progression rate. All of them were immediately treated with vitamins and CoQ10 following the diagnosis, although recovery of vision was experienced over a period of more than 4 months, with remarkable enhancement of the visual field while maintaining the prescribed treatment, which included daily CoQ10 supplementation, therefore the observed improvement in the visual field was not a result of either compensation or rehabilitation therapy. Both embolic and thrombotic occlusions of the ophthalmic artery resulting in ischemia are accompanied by a poor prognosis, even if the blockage is removed. Therefore, the observed VFI progression rates may be related to the proposed supplementation treatment. Regarding homonymous hemianopia or quadrantanopia patients following stroke (*n* = 10), the VFI progression rates were notably higher in both eyes after treatment. None of these patients received restorative therapy, including optical aids or compensatory training, and therefore the observed improvement in the visual field was not a result of rehabilitation therapy. Other patients with conditions affecting the vascularity of the retina (*n* = 13) showed similar VFI progression rates, with one experiencing full recovery of the visual field (OC04), while another (OC09) showed an increase of VFI only when CoQ10 and vitamin supplementation was prescribed at month 47 of follow-up.

**Figure 4:**
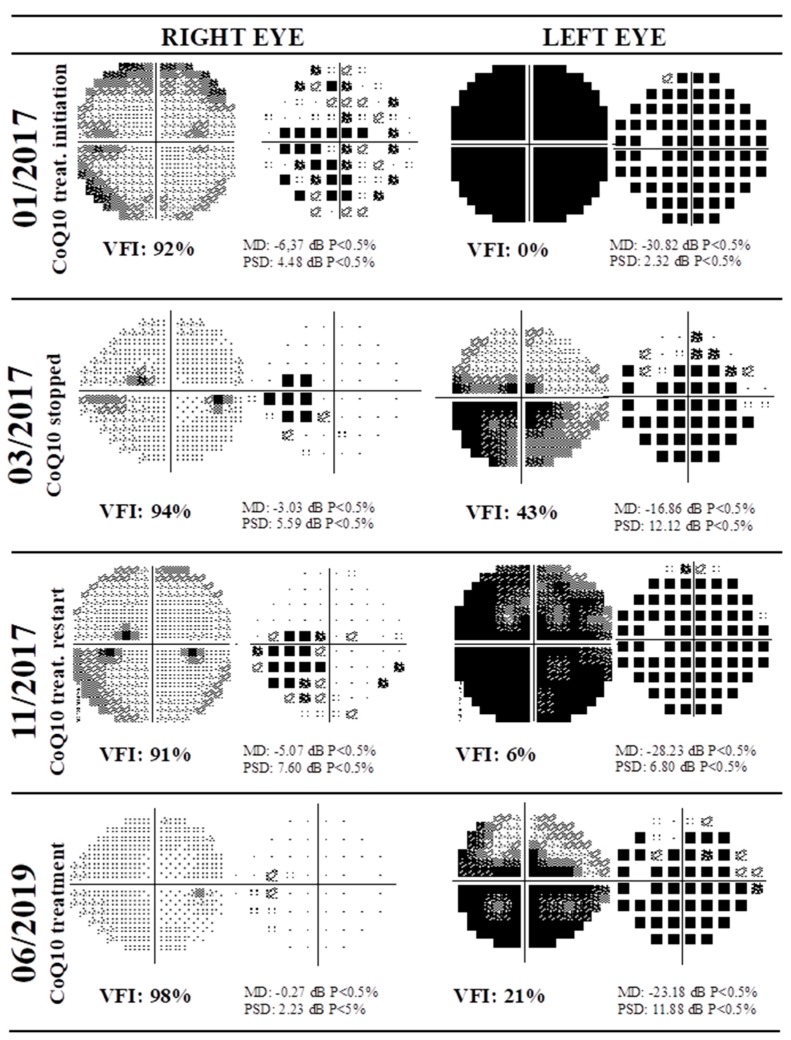
Visual field test using Humphrey field analyzer and 30-2 algorithm of a patient diagnosed with NAION (NAION02) from 2017 to 2019. Rows represent visual fields of each eye. In January 2017 the patient presented NAION in left eye, and received CoQ10 treatment. In March 2017 the CoQ10 treatment was interrupted, with deterioration in the visual field in November 2017; treatment was restarted, with observed improvement in the visual field index in June 2019.

Moreover, follow-up of NAION cases (*n* = 18) showed increased progression rates, similar to those of RAO cases, following supplementation treatment. Very interesting was patient NAION09, whose left eye VFI remained almost constant during 64 months of follow-up without treatment with CoQ10. However, from the 64th month, the patient was treated with CoQ10, showing a VFI of 55% in month 106, with an average progression rate of +2.3 ± 2, which may imply the effectiveness of this coenzyme in vision restoration. Interestingly, Figure 4 shows the visual field test using the HFA and 30-2 algorithm of NAION02 from 2017 to 2019. The patient received CoQ10 treatment after the initial examination (month 0), stopped in the third month, and restarted in month 10. In the figure, the rows represent the visual fields of each eye, showing that the left eye was dramatically affected. Upon initial treatment with CoQ10, the patient experienced recovery of the VFI from 0% to 43% in the left eye (March 2017). However, after treatment was interrupted, deterioration in the visual field to 6% was observed in November 2017. Finally, following resumption of treatment, improvement of the VFI to 21% was observed (June 2019). 

This observation is crucial, since the natural history of visual field recovery is fundamental when evaluating claims of improvement by potential treatments of vascular diseases affecting the retina [32]. We demonstrate that discontinuing treatment, with worsening VFI, and rechallenging it, with remarkable improvement in the visual field, supports the role of CoQ10 as a therapeutic agent for vascular diseases affecting the retina. Moreover, since the interruption of CoQ10 decreases the visual field, it may suggest that the beneficial effects are not irreversible, but this observation needs further confirmation. 

CoQ10, also known as ubiquinone, ubidecarenone, or coenzyme Q, is a 1,4-benzoquinone and the most common coenzyme Q in humans. CoQ10 is an essential component in mitochondrial bioenergetics, acting as an intracellular antioxidant and protecting neuronal cells against oxidative stress in neurodegenerative diseases [21,33,34]. It has been hypothesized that CoQ10 may inhibit microglial cell activation, maintaining its mitochondrial function, and prevent glutamate-induced cytotoxicity, which may contribute to neural degeneration [35]. The levels of CoQ10 may be depleted during an acute event, leading to alterations in mitochondrial energy production and increased free radical damage due to the reduced scavenging capacity [20]. 

Oral administration of CoQ10 showed neuroprotective effects in neurodegenerative diseases, including Parkinson’s disease and age-related macular degeneration [18,36], as well as in cardiovascular diseases, in which it might ameliorate endothelial dysfunction [37]. The dual role of this enzyme may contribute to its restraining extracellular glutamate accumulation and excitotoxicity, reducing the harmful effect of ischemia/reperfusion on mitochondrial energy metabolism [38]. Overall, CoQ10 has been proposed as a neurotherapeutic agent, but additional multicenter studies on the potential usefulness of its supplementation with conventional therapy in neurological diseases are needed [39,40]. Nevertheless, this study has several limitations that must be highlighted, including the absence of a control group treated with placebo and the simultaneous treatment with CoQ10 and vitamins, hindering the determination of whether the beneficial effects are due to CoQ10 alone or its combination with vitamins. 

## 5. Conclusions

In conclusion, a case series study of patients diagnosed with retinal dysfunctions caused by vascular disorders demonstrated the therapeutic potential of CoQ10 in combination with vitamins, with important improvements in the visual field. However, oral CoQ10 treatment must be evaluated in randomized, double-blind, controlled, prospective clinical studies to support these findings, in which dosing accuracy, the duration of therapy, and the effect of CoQ10 alone or combined with vitamins should be addressed. The use of CoQ10 in clinical practice together with traditional treatments can lead to improved patient outcomes.

## Figures and Tables

**Table 1:** Demographic characteristics of patients.

Study Population (n)	Age at Diagnosis (mean ± SD)	Current Age (mean ± SD)	Age Range	Gender (Female/Male)
NAION (18)	61 ± 8	61 ± 7	50–81	8(44%)/10
Stroke (10)	56 ± 21	63 ± 18	19–71	4(40%)/6
RAO (7)	65 ± 11	65 ± 11	48–83	2(29%)/5
OC (13)	47 ± 23	51 ± 22	15–85	8(61%)/5
Population-averaged (48)	57 ± 16	60 ± 15	15–85	22(46%)/26

SD, standard deviation; NAION, non-arteritic ischemic optic neuropathy; stroke, homonymous hemianopia or quadrantanopia following occipital lobe stroke; RAO, retinal artery occlusion; OC, other conditions.

**Table 2:** Clinical characteristics of NAION cases, VFI and MD follow-up, and progression rate.

Case	Disease	Follow-Up Months	Eye	Initial VFI (%)	Final VFI (%)	Initial MD (dB)	Final MD (dB)	VFI Progression Rate (%/year) ^1^
NAION01	NAION (with secondary optic atrophy)	91 ^2^	Right	96	100	−3.91	1.03	−0.2 ± 0.2
			Left	49	67	−17.91	−10.68	+2.0 ± 1.9
NAION02	NAION	29 ^3^	Right	92	98	−6.37	−0.27	+2.6 ± 2.6
			Left	0	21	−30.82	−23.18	+5.0 ± 14.8
NAION03	NAION (with homonymous hemianopia)	37 ^2^	Right	97	98	−2.4	−1.81	+1.1 ± 8.2
			Left	81	88	−6.79	−2.35	+2.4 ± 8.6
NAION04	NAION	5 ^2^	Right	97	99	−3.72	−1.52	+1.0 ± 1.1
			Left	14	35	−27.39	−21.64	+58.1 ± 10.5
NAION05	NAION	10 ^2^	Right	88	93	−5.7	−3.0	+2.8 ± 5.0
			Left	36	45	−19.22	−15.67	+11.8 ± 17.6
NAION06	NAION (with inferior hemianopia)	64 ^2^	Right	63	88	−16.43	−6.62	+5.9 ± 2.6
			Left	97	100	−1.24	0.67	−0.1 ± 0.3
NAION07	NAION	31 ^2^	Right	52	67	−17.88	−12.38	+6.0 ± 14.4
			Left	98	99	−1.48	−0.63	+0.60 ± 0.6
NAION08	NAION	14 ^2^	Right	46	79	−16.91	−6.68	+19.7 ± 13.9
			Left	93	99	−4.07	−0.13	+2.1 ± 2.6
NAION09	NAION	106 ^4^	Right	20	55	−23.96	−15.03	+2.3 ± 2.1
			Left	98	99	−2.38	−0.02	+0.0 ± 0.1
NAION10	NAION	76 ^2^	Right	99	99	−1.33	−0.28	0.0 ± 0.3
			Left	46	46	−17.9	−17.47	+0.7 ± 4.0
NAION11	NAION	12 ^2^	Right	97	99	−0.34	0.39	+0.7 ± 1.1
			Left	14	27	−27.15	−23.73	+24.5 ± 6.7
NAION12	NAION	42 ^2^	Right	78	94	−7.76	−2.15	+1.4 ± 6.8
			Left	17	25	−28.56	−26.27	+2.0 ± 4.1
NAION13	NAION	41 ^2^	Right	98	98	−0.53	1.69	+0.0 ± 0.0
			Left	56	85	−13.3	−5.26	+23.2 ± 14.5
NAION14	NAION	18 ^2^	Right	4	20	−29.64	−25.05	+14.2 ± 10.98
			Left	100	97	−2.26	−1.61	+0.1 ± 6.3
NAION15	NAION	89 ^2^	Right	67	80	−11.05	−5.96	+0.7 ± 1.2
			Left	100	99	−0.77	−0.39	−0.1 ± 0.2
NAION16	NAION	5 ^2^	Right	98	99	−0.27	1.3	+0.50 ± 0.1
			Left	78	91	−10.03	−5.02	+8.0 ± 6.6
NAION17	NAION	22 ^2^	Right	89	95	−8.69	−5.45	+1.6 ± 0.3
			Left	3	73	−30.68	−10.16	+26.6 ± 16.6
NAION18	NAION	102 ^2^	Right	52	62	−17.03	−12.1	−0.7 ± 2.0
			Left	100	99	−2.11	−1.34	+0.2 ± 0.6

^1^ 95% confidence level. ^2^ Initiation of CoQ10 treatment in month 0. ^3^ Initiation of CoQ10 treatment on day 0, stopped in month 3, and reinitiated in month 10. ^4^ Initiation of CoQ10 treatment in month 64.

**Table 3:** Clinical characteristics of homonymous hemianopia and quadrantanopia cases following stroke, VFI and MD follow-up, and progression rate.

Case	Disease	Follow-up Months	Eye	Initial VFI (%)	Final VFI (%)	Initial MD (dB)	Final MD (dB)	VFI Progression Rate (%/year) ^1^
Stroke01	Inferior homonymous hemianopia following stroke	203 ^2^	Right	55	73	−16.8	−11.43	+0.6 ± 0.9
			Left	56	78	−16.31	−9.0	+0.8 ± 0.8
Stroke02	Left homonymous hemianopia following right retrochiasmal lesion	6 ^3^	Right	60	73	−14.01	−10.47	+22.2 ± 51.1
			Left	71	66	−8.65	−9.31	−10.5 ± 5.1
Stroke03	Right superior homonymous hemianopia following left inferior retrochiasmal lesion	19 ^4^	Right	84	84	−7.72	−7.61	−0.1 ± 10.1
			Left	78	80	−10.01	−10.54	+0.7 ± 18.1
Stroke04	Right hemianopia (unknown origin)	20 ^5^	Right	51	99	−17.21	−1.18	+30.3 ± 6.4
			Left	99	100	−2.37	−0.58	+2.2 ± 4.2
Stroke05	Right inferior homonymous quadrantanopia (left superior retrochiasmal lesion)	13 ^3^	Right	77	81	−11.21	−9.94	+2.6 ± 15.7
			Left	62	90	−14.01	−4.28	+20.5 ± 14.4
Stroke06	Right homonymous incomplete hemianopia (left occipital lobe stroke)	27 ^3^	Right	72	76	−12.57	−6.92	+6.9 ± 8.6
			Left	58	71	−16.09	−12.77	+5.6 ± 1.9
Stroke07	Stroke (cerebrovascular ictus) with peripheral alteration	22 ^6^	Right	96	94	−3.34	−2.53	+3.3 ± 3.8
			Left	90	88	−5.97	−4.31	+4.9 ± 4.6
Stroke08	Left inferior homonymous quadrantanopia (right superior retrochiasmal lesion)	90 ^7^	Right	88	91	−5.62	−1.55	+2.3 ± 3.1
			Left	89	90	−5.36	−5.24	+3.1 ± 3.8
Stroke09	Right homonymous hemianopia (left retrochiasmal lesion)	12 ^3^	Right	56	65	−14.34	−10.2	+7.7 ± 5.2
			Left	50	57	−16.84	−15.31	+3.8 ± 23.8
Stroke10	Left homonymous hemianopia (right retrochiasmal lesion)	12 ^3^	Right	27	48	−22.53	−18.96	+33.3 ± 10.7
			Left	26	35	−21.28	−17.62	+16.0 ± 4.5

^1^ 95% confidence level. ^2^ Initiation of CoQ10 treatment in month 195. ^3^ Initiation of CoQ10 treatment in month 0. ^4^ Initiation of CoQ10 treatment in month 10. ^5^ Initiation of CoQ10 treatment in month 4. ^6^ Stroke in third month and CoQ10 in 15th month. ^7^ Initiation of CoQ10 treatment in month 68.

**Table 4:** Clinical characteristics of RAO patients, VFI and MD follow-up, and progression rate.

Case	Disease	Follow-Up Months	Eye	Initial VFI (%)	Final VFI (%)	Initial MD (dB)	Final MD (dB)	VFI Progression Rate (%/year) ^1^
RAO01	CRAO	20 ^2^	Right	0	75	−31.24	−10.19	+39.9 ± 25.6
			Left	98	99	−1.27	0.94	+1.8 ± 4.0
RAO02	CRAO	9 ^2^	Right	0	46	−31.88	−17.3	+49.4 ± 42.9
			Left	96	94	−3.15	−2.19	+1.0 ± 10.1
RAO03	RAO (temporal inferior with nasal superior quadrantanopia)	4 ^2^	Right	100	96	0.9	−2.37	−2.0 ± 1.9
			Left	48	78	−18.55	−8.06	+27.5 ± 12.7
RAO04	RAO (temporal inferior with optic nerve atrophy)	7 ^2^	Right	98	100	−0.57	0.7	+1.0 ± 0.8
			Left	62	78	−10.79	−7.04	+12.2.5 ± 10.6
RAO05	RAO (temporal inferior with superior hemianopia)	15 ^2^	Right	47	87	−15.45	−5.71	+13.1 ± 16.9
			Left	98	99	−3.17	−1.99	+0.2 ± 0.2
RAO06	RAO (temporal superior)	5 ^2^	Right	56	61	−14.44	−11.09	+8.9 ± 3.5
			Left	98	98	−2.27	−1.54	+0.0 ± 0.0
RAO07	RAO (temporal superior)	53 ^2^	Right	95	98	−2.5	−0.39	+0.2 ± 1.1
			Left	51	61	−18.3	−12.68	+3.0 ± 5.3

^1^ 95% confidence level. ^2^ Initiation of CoQ10 treatment in month 0. CRAO, central retinal artery occlusion.

**Table 5:** Clinical characteristics of patients with other conditions, VFI and MD follow-up, and progression rate.

Case	Disease	Follow-Up Months	Eye	Initial VFI (%)	Final VFI (%)	Initial MD (dB)	Final MD (dB)	VFI Progression Rate (%/year) ^1^
OC01	Optic neuritis	92 ^2^	Right	87	97	−9.54	−2.61	+0.9 ± 0.5
			Left	71	94	−9.89	−3.19	−2.4 ± 0.6
OC02	Optic neuritis causing optic nerve atrophy	97 ^2^	Right	99	98	−1.44	−2.22	−0.4 ± 0.4
			Left	70	91	−11.12	−7.06	+0.8 ± 3.5
OC03	Bilateral optic neuritis	16 ^2^	Right	48	92	−20.89	−5.65	+24.2 ± 28.2
			Left	22	93	−27.32	−3.94	+61.7 ± 32.9
OC04	Optic nerve atrophy (post-meningitis)	63 ^2^	Right	0	11	−31.02	−25.79	+76.2 ± 12.3
			Left	2	91	−30.22	−2.4	+257.0 ± 42.30
OC05	Optic nerve atrophy (intracranial hypertension)	110 ^2^	Right	89	85	−0.87	−7.12	+1.3 ± 3.8
			Left	89	88	1.15	−6.72	+0.6 ± 2.4
OC06	Optic nerve atrophy (drug toxicity, etambutol)	22 ^2^	Right	86	99	−4.58	−0.28	+4.2 ± 2.4
			Left	87	99	−5.67	−0.1	+7.1 ± 3.0
OC07	Neuroretinitis	26 ^2^	Right	70	88	−11.47	−2.31	+6.8 ± 5.4
			Left	100	100	4.06	4.18	+0.1 ± 0.4
OC08	Tapetoretinal dystrophy (with superior hemianopia)	34 ^2^	Right	31	40	−26.17	−20.8	+2.2 ± 2.9
			Left	22	48	−27.64	−18.42	+2.5 ± 3.9
OC09	Retinitis pigmentosa	53 ^3^	Right	12	49	−27.92	−19.46	+0.0 ± 3.7
			Left	19	49	−26.88	−19.85	+0.4 ± 2.7
OC10	Unknown	14 ^2^	Right	5	80	−28.75	−7.54	+15.5 ± 4.4
			Left	11	88	−27.76	−8.5	+10.0 ± 4.9
OC11	Unknown (optic nerve injury)	68 ^4^	Right	95	98	−5.13	−1.9	+4.1 ± 24.6
			Left	90	99	−6.4	−0.34	+4.6 ± 22.0
OC12	Cone dystrophy	103 ^5^	Right	66	70	−6.99	−9.96	+0.1 ± 1.7
			Left	71	70	−7.39	−10.0	−0.6 ± 1.1
OC13	Retinal vascular occlusion	89 ^6^	Right	99	99	−0.22	−0.51	+0.6 ± 1.7
			Left	31	58	−24.44	−17.84	+5.2 ± 2.9

^1^ 95% confidence level. ^2^ Initiation of CoQ10 treatment in month 0. ^3^ Initiation of CoQ10 treatment in month 47. ^4^ Initiation of CoQ10 treatment in month 40. ^5^ Initiation of CoQ10 treatment in month 93. ^6^ Initiation of CoQ10 treatment in month 57.

**Table 6:** P-values obtained for comparisons between initial and final values of VFI (%) and MD (db) of each group of patients.

*p*-Values (Wilcoxon Matched-Pairs Test)		
Group	VFI (Initial vs. Final) (%)	MD (Initial vs. Final) (db)
NAION	< 0.0001	< 0.0001
Stroke	0.0008	< 0.0001
RAO	0.0081	0.0023
OC	< 0.0001	0.0005

NAION, non-arteritic ischemic optic neuropathy; Stroke, homonymous hemianopia or quadrantanopia following occipital lobe stroke; RAO, retinal artery occlusion; OC, other conditions.

## Supplementary Materials

The following are available online at https://www.mdpi.com/2072-6643/12/3/723/s1, Table S1: Demographic data and clinical conditions.

## References

[1] Hayreh S.S. (2009). Ischemic optic neuropathy. Prog. Retin. Eye Res..

[2] Buono L.M., Foroozan R., Sergott R.C., Savino P.J. (2002). Nonarteritic anterior ischemic optic neuropathy. Curr. Opin. Ophthalmol..

[3] Varma D.D., Cugati S., Lee A.W., Chen C.S. (2013). A review of central retinal artery occlusion: Clinical presentation and management. Eye.

[4] Limaye K., Wall M., Uwaydat S., Ali S., Shaban A., Al Kasab S., Adams H. (2018). Is Management of Central Retinal Artery Occlusion the Next Frontier in Cerebrovascular Diseases?. J. Stroke Cerebrovasc. Dis..

[5] Hayreh S.S. (1995). The 1994 Von Sallman Lecture. The optic nerve head circulation in health and disease. Exp. Eye Res..

[6] Goodwin D. (2014). Homonymous hemianopia: Challenges and solutions. Clin. Ophthalmol..

[7] Eggenberger E.R., Pula J.H., Aminoff M.J., Josephson S.A. (2014). Neuro-ophthalmology in Medicine. Aminoff’s Neurology and General Medicine.

[8] Hayreh S.S., Zimmerman M.B., Podhajsky P., Alward W.L.M. (1994). Nocturnal arterial hypotension and its role in optic nerve head and ocular ischemic disorders. Am. J. Ophthalmol..

[9] Hayreh S.S. (1996). Acute ischemic disorders of the optic nerve: Pathogenesis, clinical manifestations and management. Ophthalmol. Clin. N. Am..

[10] Hayreh S.S. (2012). Non-arteritic anterior ischemic optic neuropathy versus cerebral ischemic stroke. Graefes Arch. Clin. Exp. Ophthalmol..

[11] Pula J.H., Yuen C.A. (2017). Eyes and strike: The visual aspects of cerebrovascular disease. Stroke Vasc. Neurol..

[12] Hayreh S.S. (2001). Blood flow in the optic nerve head and factors that may influence it. Prog. Retin. Eye Res..

[13] Pambakian A., Currie J., Kennard C. (2005). Rehabilitation strategies for patients with homonymous visual field defects. J. Neuroophthalmol..

[14] Brown N.A., Bron A.J., Harding J.J., Dewar H.M. (1998). Nutrition supplements and the eye. Eye (Lond.).

[15] Demmig-Adams B., Adams R.B. (2013). Eye Nutrition in Context: Mechanisms, Implementation, and Future Directions. Nutrients.

[16] Salama M., Yuan T.F., Machado S., Murillo-Rodríguez E., Vega J.A., Menéndez-González M., Nardi A.E., Arias-Carrión O. (2013). Co-enzyme Q10 to treat neurological disorders: Basic mechanisms, clinical outcomes, and future research direction. CNS Neurol. Disord. Drug Targets.

[17] Zhang X., Biswas L., Tohari A.M., Reilly J., Tiano L., Shu X. (2017). Coenzyme Q10 as a therapeutic candidate for treating inherited photoreceptor degeneration. Neural Regen. Res..

[18] Zhang X., Tohari A.M., Marcheggiani F., Zhou X., Reilly J., Tiano L., Shu X. (2017). Therapeutic Potential of Co-enzyme Q10 in Retinal Diseases. Curr. Med. Chem..

[19] Martucci A., Nucci C. (2019). Evidence on neuroprotective properties of coenzyme Q10 in the treatment of glaucoma. Neural Regen. Res..

[20] Yang X., Zhang Y., Xu H., Luo X., Yu J., Liu J., Chang R.C. (2016). Neuroprotection of Coenzyme Q10 in Neurodegenerative Diseases. Curr. Top. Med. Chem..

[21] Somayajulu M., McCarthy S., Hung M., Sikorska M., Borowy-Borowski H., Pandey S. (2005). Role of mitochondria in neuronal cell death induced by oxidative stress; neuroprotection by coenzyme Q10. Neurobiol. Dis..

[22] Fernández-Vega B., González-Iglesias H., Vega J.A., Nicieza J., Fernández-Vega A. (2018). Coenzyme Q10 treatment improved visual field after homonymous quadrantanopia caused by occipital lobe infarction. Am. J. Ophthalmol. Case Rep..

[23] Prem Senthil N., Khadka J., Gilhotra J.S., Simon S., Fenwick E.K., Lamoureux E., Pesudovs K. (2019). Understanding quality of life impact in people with retinal vein occlusion: A qualitative inquiry. Clin. Exp. Optom..

[24] Kerkhoff G. (1999). Restorative and compensatory therapy approaches in cerebral blindness—A review. Restor. Neurol. Neurosci..

[25] Augsburger J.J., Magargal L.E. (1980). Visual prognosis following treatment of acute central retinal artery obstruction. Br. J. Ophthalmol..

[26] Miller N.J., Arnold A.C. (2015). Current concepts in the diagnosis, pathogenesis and management of nonarteritic anterior ischaemic optic neuropathy. Eye (Lond.).

[27] Pambakian A.L., Kennard C. (1997). Can visual function be restored in patients with homonymous hemianopia?. Br. J. Ophthalmol..

[28] Scherer R.W., Feldon S.E., Levin L., Langenberg P., Katz J., Keyl P.M., Wilson P.D., Kelman S.E., Dickersin K. (2008). Ischemic Optic Neuropathy Decompression Trial Research Group. Visual fields at follow-up in the Ischemic Optic Neuropathy Decompression Trial: Evaluation of change in pattern defect and severity over time. Ophthalmology.

[29] Frolov A., Feuerstein J., Subramanian P.S. (2017). Homonymous Hemianopia and Vision. Restoration Therapy. Neurol. Clin..

[30] Mirshahi A., Feltgen N., Hansen L.L., Hattenbach L.O. (2008). Retinal vascular occlusions: An interdisciplinary challenge. Dtsch. Arztebl. Int..

[31] Han L., Law-Gibson D., Reding M. (2002). Key neurological impairments influence function-related group outcomes after stroke. Stroke.

[32] Kedar S., Ghate D., Corbett J.J. (2011). Visual fields in neuro-ophthalmology. Indian J. Ophthalmol..

[33] Litarru G.P., Tiano L. (2007). Bioenergetic and antioxidant properties of coenzyme Q10: Recent developments. Mol. Biotechnol..

[34] Lee D., Kim K.Y., Shim M.S., Kim S.Y., Ellisman M.H., Weinreb R.N., Ju W.K. (2014). Coenzyme Q10 ameliorates oxidative stress and prevents mitochondrial alteration in ischemic retinal injury. Apoptosis.

[35] Lu C.J., Guo Y.Z., Zhang Y., Yang L., Chang Y., Zhang J.W., Jing L., Zhang J.Z. (2017). Coenzyme Q10 ameliorates cerebral ischemia reperfusion injury in hyperglycemic rats. Pathol. Res. Pract..

[36] Beal M.F. (2004). Therapeutic effects of coenzyme Q10 in neurodegenerative diseases. Methods Enzymol..

[37] Molyneux A., Florkowski C.M., George P.M., Pilbrow A.P., Frampton C.M., Lever M., Richards A.M. (2008). Coenzyme Q10: An independent predictor of mortality in chronic heart failure. J. Am. Coll. Cardiol..

[38] Nucci C., Tartaglione R., Cerulli A., Mancino R., Spanò A., Cavaliere F., Rombolà L., Bagetta G., Corasaniti M.T., Morrone L.A. (2007). Retinal damage caused by high intraocular pressure-induced transient ischemia is prevented by coenzyme Q10 in rat. Int. Rev. Neurobiol..

[39] Russo R., Cavaliere F., Rombolà L., Gliozzi M., Cerulli A., Nucci C., Fazzi E., Bagetta G., Corasaniti M.T., Morrone L.A. (2008). Rational basis for the development of coenzyme Q10 as a neurotherapeutic agent for retinal protection. Prog. Brain Res..

[40] Hernández-Camacho J.D., Bernier M., López-Lluch G., Navas P. (2018). Coenzyme Q10 Supplementation in Aging and Disease. Front. Physiol..

